# Integration of eHealth Tools in the Process of Workplace Health Promotion: Proposal for Design and Implementation

**DOI:** 10.2196/jmir.8769

**Published:** 2018-02-23

**Authors:** Paulino Jimenez, Anita Bregenzer

**Affiliations:** ^1^ Department of Psychology University of Graz Graz Austria

**Keywords:** eHealth, health promotion, mHealth, occupational health, workplace

## Abstract

**Background:**

Electronic health (eHealth) and mobile health (mHealth) tools can support and improve the whole process of workplace health promotion (WHP) projects. However, several challenges and opportunities have to be considered while integrating these tools in WHP projects. Currently, a large number of eHealth tools are developed for changing health behavior, but these tools can support the whole WHP process, including group administration, information flow, assessment, intervention development process, or evaluation.

**Objective:**

To support a successful implementation of eHealth tools in the whole WHP processes, we introduce a concept of WHP (life cycle model of WHP) with 7 steps and present critical and success factors for the implementation of eHealth tools in each step.

**Methods:**

We developed a life cycle model of WHP based on the World Health Organization (WHO) model of healthy workplace continual improvement process. We suggest adaptations to the WHO model to demonstrate the large number of possibilities to implement eHealth tools in WHP as well as possible critical points in the implementation process.

**Results:**

eHealth tools can enhance the efficiency of WHP in each of the 7 steps of the presented life cycle model of WHP. Specifically, eHealth tools can support by offering easier administration, providing an information and communication platform, supporting assessments, presenting and discussing assessment results in a dashboard, and offering interventions to change individual health behavior. Important success factors include the possibility to give automatic feedback about health parameters, create incentive systems, or bring together a large number of health experts in one place. Critical factors such as data security, anonymity, or lack of management involvement have to be addressed carefully to prevent nonparticipation and dropouts.

**Conclusions:**

Using eHealth tools can support WHP, but clear regulations for the usage and implementation of these tools at the workplace are needed to secure quality and reach sustainable results.

## Introduction

### Workplace Health Promotion

Workplace health promotion (WHP) projects in organizations are one of the key solutions for improving health in organizations [1]. WHP projects include the whole package of analyzing the current structures and procedures in the organization, developing interventions for individuals to support them to change their own health behavior, and developing interventions for the organization to change critical working conditions. Electronic health (eHealth) and mobile health (mHealth) tools could be used to support and to improve the whole process of WHP projects. On the other hand, there is much uncertainty about the role of eHealth and mHealth tools in organizations, especially when it comes to health-relevant data assessed within a WHP process. Therefore, there are several points to be considered about the challenges and opportunities, which lie in this approach.

eHealth refers to the use of technology (mostly including the Internet) in health-related services, whereas mHealth includes mobile and wireless technologies (eg, mobile phone apps, wearable devices) in health programs [2]. mHealth can be seen as a specific part of eHealth; therefore, we use the term eHealth as an umbrella term for using electronic or mobile devices for health services.

eHealth tools can raise interest, motivation, and participation in WHP projects [3,4]. Especially the point of participation is one of the major issues in today’s WHP projects, as the majority of participants in WHP projects consist of a selected group of people with healthier lifestyles [5]. eHealth tools can support raising participation quotes of employees in WHP projects, as they are able to help employees overcome such barriers [6]. Indeed, studies have reported that eHealth tools seem to be more attractive for unhealthier employees, as they provide the possibility to stay anonymous [7,8].

Another issue in WHP is ineffective planning of the WHP process and the tendency to develop interventions that try to reach the broadest range of employees (“one-fits-all-principle”). This might be an obstacle for employees to participate in WHP projects, as a broad content of WHP interventions might not sufficiently meet the demands of the specific target group. Tailored WHP projects specifically developed for each organization have shown to raise participation quotes in WHP projects [9]. Especially tailored feedback is an important motivator for the employees’ participation in WHP programs [8]. This is where eHealth tools can bring considerable added value as these tools can be easily programmed to meet the demands of the individual user (eg, by giving instant, tailored feedback).

Currently, a large number of eHealth tools are available in all areas of health promotion—including applications for sports, weight reduction, and healthy nutrition [10] as well as applications addressing psychological factors (eg, to reduce stress or burnout, enhance recovery and coping strategies, or learn new competencies and skills) [11]. Current eHealth tools focus strongly on changing individual attitudes and behavior, but rarely focus on the improvement of the working environment such as analyzing and changing working conditions (an exception is presented by Koldijk et al [11]). Supporting the whole WHP process (including the analysis of the current state, intervention development process, intervention implementation, and further activities) is even less addressed in the currently developed eHealth tools. Therefore, solutions in the field of eHealth for WHP should focus more strongly on targeting the whole WHP process, especially on improving the working environment.

In addition, the usage of eHealth tools should be regulated similar to any other psychotherapeutic or medical intervention [12]. Therefore, eHealth tools in WHP should fulfill certain quality criteria to be trusted and accepted by an organization and its employees. Quality criteria mainly concern functionality, aesthetics, and security of eHealth tools [13,14], but can also target feedback systems or communication of results [12,15].

Combining eHealth tools and the current WHP processes can add sustainability by enhancing motivation and interest, as noted. The necessity to find rules for the successful implementation and having a guideline to avoid some pitfalls, on the other hand, emerged during different studies where the authors were asked to evaluate and support projects in WHP. The possibilities of combining eHealth tools and WHP were discussed in these projects with health experts from different fields (psychologists, physicians, nutritionists, kinesiologists, and other experts). These steps led to small pilot projects, in which eHealth tools were applied to the working context. The discussions and pilot projects provided a good basis for the further development of a WHP process model that allows integrating eHealth tools on different levels.

In this paper, we introduce a concept of WHP that supports the design and integration of eHealth tools in each step of the WHP process and present success factors and possible obstacles for the implementation. The presented concept is based on several models, especially the “WHO model of healthy workplace continual improvement process” [16], the models and criteria of the European Network for Workplace Health Promotion [17,18], the criteria of the International Labour Organization [19], and specifications such as the DIN SPEC 91020 [20]. We suggest adaptations to these models to allow a smooth integration of eHealth tools in the WHP process. Furthermore, we provide recommendations in the form of guidelines on how to implement eHealth tools for WHP in the practical field.

### Policies and Strategies for Workplace Health Promotion

WHP can be defined as “the combined efforts of employers, employees and society to improve the health and well-being of people at work” [20]. The Luxembourg Declaration on Workplace Health Promotion [21]—which is generally used as a framework for planning and executing WHP projects—has established guidelines that have to be fulfilled for successful WHP projects:

a) All staff have to be involved (participation), b) WHP has to be integrated in all important decisions and in all areas of organisations (integration), c) all measures and programs have to be oriented to a problem-solving cycle: needs analysis, setting priorities, planning, implementation, continuous control and evaluation (project management), and d) WHP includes individual-directed and environmental-directed measures from various fields. It combines the strategy of risk reduction with the strategy of the development of protection factors and health potentials (comprehensiveness).

Especially the last point of these guidelines about combining individual-focused and organization-focused strategies is not always considered in the practical field. The individual-focused approach includes aspects such as coping and time-management skills [22], or fitness activities and lifestyle guidance [23]. In the organization-focused approach, the work environment and the modification of the work conditions and work structures are addressed [23,24]. Interventions in the organization-focused approach include clarifying job designs [24], changing working hours, reducing shift work and unpredictable working hours, encouraging flexible work arrangements [22,24,25], and introducing supportive leadership styles and a supportive and comfortable social climate [23].

In the majority of WHP projects, individual-focused interventions are conducted and the organization-focused method is less addressed [26]. However, pursuing the organization-focused approach is more sustainable and can have a much broader impact on the employees’ health than only focusing on reducing individual risk factors [27].

### A Comprehensive Perspective on Workplace Health Promotion

We present a WHP process with 7 steps that can assist organizations in conducting successful WHP projects. We suggest adaptations to these models to allow a smooth integration of eHealth tools in the WHP process. Categorizing the process in steps is important, as every step can contain facilitating and hindering factors that need to be addressed [28]. Furthermore, planning WHP projects in individual steps can support the responsible persons in organizing and deploying the needed resources for each step [29] and support a successful implementation of eHealth tools in the WHP process. To discover the possibilities and benefits of including eHealth tools in WHP, we need to understand the aim of each step and we need to identify critical factors that might prevent reaching these aims successfully.

The most holistic WHP process has been established by the World Health Organization (WHO) [16]. In their “WHO model of healthy workplace continual improvement process,” 8 steps are defined in a circle, indicating that WHP is an ongoing process that develops and improves over time. The WHO model shares similarities with other models [30-32] that present steps to successfully conduct employee surveys to assess job demands and job resources. In these models, the first step always comprises the conceptual design of the WHP process, which is indirectly included in the WHO model in the steps “mobilize” and “assemble.” Another step includes the analysis as well as the presentation and interpretation of the analysis results, which is included in the WHO model under the steps “assess” and “prioritize.” However, assessment and presentation of the assessment results should be distinguished, as the presentation of the assessment results usually comprises discussions with the management [32]. These discussions further lead to the development of an intervention implementation plan [31]. Therefore, especially when using eHealth tools, the presentation step should be treated separately to the actual assessment to implement the right tools for the right process step and, at last, to achieve the best possible outcome.

## Implementation of eHealth Tools in Workplace Health Promotion

### Life Cycle Model of Workplace Health Promotion

**Figure 1:**
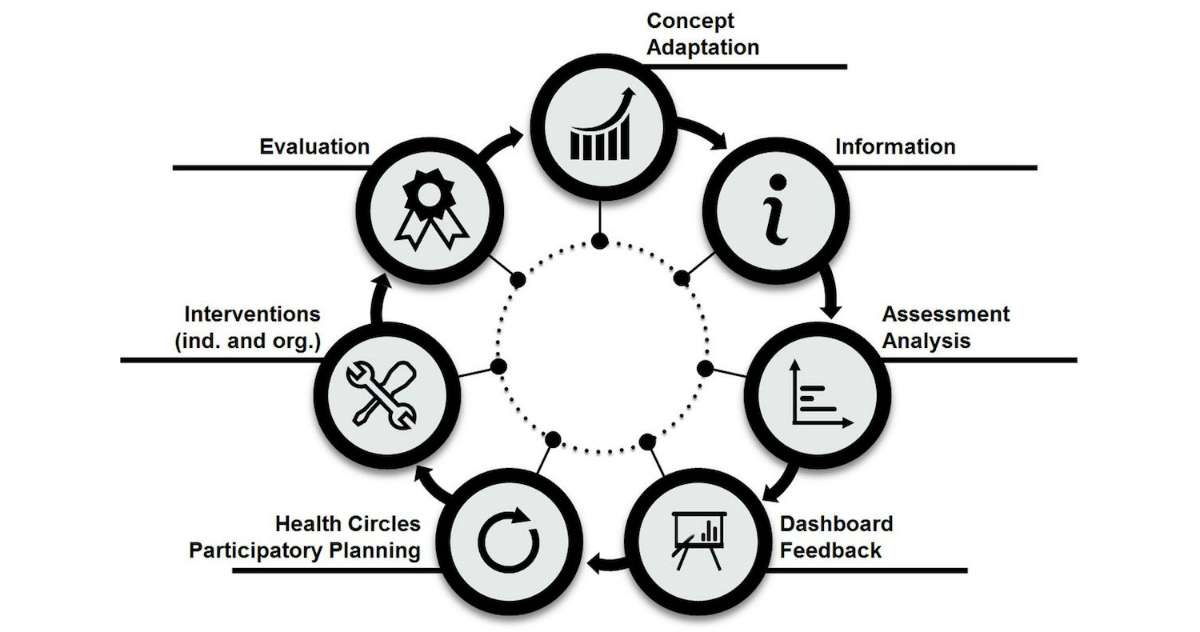
Life cycle model of workplace health promotion.

The proposed “life cycle model of Workplace Health Promotion” (Figure 1) is based on the WHO model and other models and focuses on the support of the integration of eHealth tools (Figure 1): (1) Concept/Adaptation, (2) Information, (3) Assessment/ Analysis, (4) Dashboard Feedback, (5) Health Circles/ Participatory Planning, (6) Interventions (individual/ organization), and (7) Evaluation. We first present the adapted model applicable for WHP in general and then present the important issues for the integration of eHealth tools in that process. Implementing eHealth tools can be a great advantage in every step. The content of each step as well as the advantages and possible implementation procedures for eHealth tools are described in the next chapters.

### Concept/Adaptation

The first step includes planning the WHP project (duration and content of the project, needed resources and experts) and setting the main objectives. It is also important to discuss if the project should be conducted identically in all locations and departments or if some departments or locations get a slightly different concept. These decisions can be made by the senior management or by other persons with decision authority [29]. However, for the success of the WHP project, these discussions should involve all relevant stakeholders (eg, management, human resources department, company physicians, safety specialists, work and organizational psychologists, work council, employee representatives). These key stakeholders should demonstrate their commitment to the WHP project by assembling a steering group [30,33] or a “healthy workplace team” [16]. Typically, this is the first time where key questions regarding anonymity of data, responsibilities, or data security are discussed [30]. This discussion is a core point, as critical aspects can influence all other steps and preclude the success of the whole project. Regarding eHealth tools, a plan for the correct implementation of these tools should be discussed in the steering group as well [34].

A great advantage of eHealth tools in this first step could lie in providing the opportunity to keep in contact with all relevant stakeholders. These tools can support the cooperation between the steering group and other experts involved in the WHP process by providing a platform for communication. This platform can be further used to set and track the objectives of the WHP project, to manage the information flow to the employees, to monitor the assessment of the current state of the organization and the interventions (date, duration, location, content), and to manage intervention groups (participation rates, satisfaction). Monitoring satisfaction ratings and participation rates can provide a good overview of the overall acceptability of the WHP program, and they should be assessed during the whole WHP process [35]. However, it is also important that the steering group and experts meet face-to-face as much as possible for clearer communication and discussions [15].

### Information

An important factor for success is establishing a clear information flow between the management, key stakeholders, steering group, and employees. All persons have to be informed about the project and its concept and objectives. Information should be given in a top-down process; this means the information flow should follow the hierarchical structure of the company. As this information flow is very important to “take fears” and gain the trust of the employees, this step is typically a separate step in models of WHP (see also [16]). All employees have to be informed about the whole WHP process and its different steps. This transparency ensures that the WHP project does not raise false expectations on the part of the employees that could later lead to disappointment or negative appraisal [36]. One major issue in this step concerns fears about exposure or surveillance. Employees have to be guaranteed anonymity and strict data protection. In addition, it must be clear that the participation is voluntary.

Especially mHealth tools can reach a large number of people, as they are not restricted to a certain location [37]. By offering different channels of communication (eg, text messages, audio and video files), users can be reached in every setting and situation [38]. The overall accessibility of health programs is especially suitable for programs targeting mental health, as persons with mental health problems can avoid exposure in an open group when using eHealth tools [6,39]. Having all information in one tool that is accessible at any time and in any location could help raising awareness and motivation for the activities planned in the WHP process. In addition, information should be given about the added values and benefits of using eHealth tools in WHP as well as information about how to use these tools and data security issues [37]. These tools should be introduced to the employees as nondirective as possible (eg, by letting the employees themselves decide if and how to use the tools) to avoid possible resistance [40].

Such an information board for the employees could also include the possibility to interact with health experts or the steering committee. Having social contact with other participants in the WHP program enhances participation rates and raises the effectiveness of eHealth tools [41]. Platforms for social contact could also include communication between the employees and health experts to share or discuss health issues [42].

### Assessment/Analysis

The next step is to analyze the current state of the organization in accordance with the objectives set in the first step. Usually, surveys in the form of questionnaires or interactive workshops are used as analysis tools [31]. Next to a so-called needs assessment, where the employees are asked about their wishes regarding health promotion activities, other physical and psychological factors are assessed as well (eg, health status of employees, job satisfaction, motivation, and commitment) [16]. Other resources such as key data on sickness absences or fluctuation as well as data from previous WHP projects can be valuable additions to the data obtained in surveys or workshops. Furthermore, it is important to analyze aspects of the workplace and the organization (eg, policy, rules, regulations, available resources) to be able to successfully integrate health promotion activities within the workplace environment [33].

eHealth tools can be used to assess, store, and analyze questionnaires and psychophysiological data [43]. In WHP projects, the assessment is usually very comprehensive, consisting of many questionnaires. Therefore, Web-based tools should be used instead of mobile tools, as filling questionnaires on the Web is more feasible than on mobile devices [44]. By supporting the assessment with Web tools, it becomes easier to manage data and results, as all data are immediately stored at one place and can be accessed from different locations [45]. In addition to Web tools, the mobile phone can be used to conduct short surveys or tests [46]. The assessment tool could also support in combining different data sources (eg, questionnaire data, interview data, behavioral analyses, psychophysiological data, or corporate key figures of the company). A combination of different data sources can also prevent demotivation when it comes to data collection, as collecting a lot of questionnaire data might be perceived as an additional burden for the employee [37].

### Dashboard Feedback

The interpretation of the results has to be done including experts in the field of health promotion (eg, safety officer, company physician, work and organizational psychologist). Similar to the third step, information flow, informing all persons about the analysis results must be done in a top-down process, starting with the top management and cascading down to the middle management, lower management, and employees [31]. First, the results are discussed internally in the steering group and top management. In this phase, the group interprets the results and defines certain areas of focus or task assignments [31]. Going further in the top-down process, the assessment results should be communicated in an easy language so that they are easily understood and accepted by the employees [47].

eHealth tools can support transferring the assessment results into feedback pages automatically. These feedback pages summarize the collected data and ideally present them in a clear and concise way with simple graphical representations [48]. This easy-to-read recap of the process can be called “dashboard” and can be used as a first rough indicator in addition to the more detailed analysis of the assessment. It is important that the information will be presented in ways that are meaningful, accurate, and easily understood [12]. This can be a challenge for eHealth tools, as the information gained from the data has to be presented in the best quality and tailored to the company and the user [49]. The main results in the dashboard can be unlocked for the whole company and all employees or only for specified groups or persons. However, it seems to be a good approach to provide the feedback pages to all employees to ensure further participation in the next step of intervention planning [30]. eHealth tools can especially support in a comprehensive presentation of the results. However, the interpretation of the results should be done by health experts as well as separately and outside these tools to avoid misinterpretations.

### Health Circles/Participatory Planning

After presenting and discussing the analysis results, a health plan has to be established and specific interventions have to be developed to promote health at the workplace [16]. In this step, employees should be involved in the decision-making process to raise the employees’ acceptance toward the developed interventions and increase participation rates of employees [28]. A good approach is the building of “health circles,” which are discussion groups within the organization to develop options for improving health at the workplace [50,51]. The developed interventions can address improving health either directly by offering sports activities or health-related workshops, or indirectly by changing potentially harmful work conditions (which is stated in the Luxembourg Declaration [21] as individual-focused and organization-focused measures).

With the support of eHealth tools, the organization of health circles can be improved by adding calendar options or inviting interested employees to the health circles with messages. In addition, the whole execution of the health circles can be supported by providing information about the assessment results in a dashboard and thus having access to the results at any time. The developed interventions as well as information about further steps in the process can be entered into the eHealth tool, and this information can be provided to the whole company and all employees or to specified groups or persons.

### Interventions (Individual/Organization)

In this step, the developed interventions from the health circles are implemented. Interventions should be developed with attention to health-beneficial effects, but also with special attention to organizational frameworks and individual preferences that allow high participation rates in the developed interventions. Interventions should be easy to implement and to attend (geographically and chronologically) and should be perceived as interesting and meaningful to enhance participation [52].

This is the step where the majority of the eHealth tools are currently developed. These tools can strongly support interventions, especially individual-focused interventions (eg, monitoring physical and mental health or nutrition intake, activity tracking). eHealth tools provide the possibility to combine monitoring devices (eg, tracking belts, smart watches) and WHP programs to track physical exercises or weight loss [8,53]. With these devices, psychophysiological data (eg, heart rate or heart rate variability for stress assessment) could also be assessed, and in a next step, it could be combined with self-reported questionnaire data [54]. Including instant, individual feedback developed by experts gives users additional information about their health progress and helps them to understand the meaning of the collected data [53]. Offering individualized feedback about the employees’ personal health status and health progress along with information about programs to improve health are important motivators for the employees’ participation in the WHP programs [8,9,44,55,56]. This is where eHealth tools can bring considerable added value, as these tools can be easily programmed to give instant, tailored feedback for each user. Getting feedback is part of a self-monitoring and goal-setting process that can raise the motivation for changing behavior [44].

However, it is very important that the feedback is programmed together with the knowledge and skills of experts in the field, as feedback about health parameters can contain critical information that might lead to misinterpretations [57]. This is especially the case if the feedback shows a critical health result, which might raise the participants’ fears about serious health issues [58]. In the case of stress feedback, getting critical feedback could also cause more stress [54]. Therefore, automatic feedback ideally should be given together with possibilities to talk with a health expert. Instant or delayed feedback can also be seen as incentives and can raise the motivation to participate in an intervention. Hence, the effects of a WHP project can be attributed to the sustainable participation in interventions, especially if eHealth tools are implemented [59].

With regard to organization-focused interventions, the possibilities of eHealth tools have not been used to their full potential yet. Improving the environmental conditions at the workplace (eg, the organizational structures, social climate, or management) can strongly support health at the workplace in a sustainable way [26]. However, organization-focused interventions are often difficult to carry out and to organize [60]. The implementation of organization-focused interventions has to be managed as clearly and structured as possible to achieve the most successful outcome [61]. eHealth tools can support the organization in organizing and evaluating organization-focused interventions by providing management dashboards where the process and responsibilities are managed.

### Evaluation

The step of evaluation comprises evaluating the implementation process and the implementation outcomes. To conduct an evaluation, the organization can go through steps 1-6 again, starting with the first step “Concept/Adaptation.” According to WHO [16], an evaluation should be done at least every 3-5 years. Aims of the evaluation can comprise whether the participation rates are satisfactory or whether the implemented interventions have been effective [32]. The effectiveness of an intervention can be analyzed by evaluating the proximate (short-term) outcomes (eg, improvement of individual skills), intermediate (medium-term) outcomes (eg, changes in demands and resources, social processes, leadership behavior), and distal (long-term) outcomes (eg, improvement of individual health or organizational performance), which are all important outcomes in the intervention context [62].

eHealth tools can support the whole evaluation process. The evaluation should be focused on summative (after implementation) as well as formative evaluation (throughout the whole life cycle of WHP) and can also include the evaluation of the software development cycle of the eHealth tool [63]. An advantage of eHealth tools that is often stated is that they are cheaper than “traditional” health programs (eg, [64]). Therefore, it is suggested to evaluate the cost-effectiveness of the intervention in addition to the effectiveness of the intervention [65]. An evaluation can be also done regarding the acceptance and usability of an eHealth tool, which optimally results in a continuous improvement process [49]. A tool that is perceived as useful and easy-to-use can raise the usage of the tool [42]; therefore, these aspects should be evaluated regularly.

### Organizational Requirements

#### Implementation Requirements

eHealth tools offer many possibilities and benefits, but open questions for the implementation of these tools still remain. The most commonly reported barriers (but also facilitators) of WHP projects were found in the characteristics of the organization, for example, lack of resources, no fit between intervention and organizational culture, or lack of managerial support [66]. The organizational characteristics should be investigated very carefully to ensure the success of a WHP project. This is especially the case if WHP projects are supported with eHealth tools. The complexity of the working environment could influence the way employees use eHealth tools, and thus, the work setting must be analyzed carefully before implementing these tools [67].

In the best case, the eHealth tools are fully integrated in the organizational structure and working routines [68]. Failing to integrate eHealth tools as a part of the organization’s everyday life can be a barrier that prevents employees from using these tools at the workplace [37]. A framework for the integration of eHealth tools has to consider at least the part of the employees, the targeted user group, and the organization that provides the environment for the usage of the tools.

Regarding the employees, the organization has to provide opportunities to use the eHealth tools at work, such as providing the tools (eg, access to computers and mobile phones), providing training and technical support, and providing possibilities to use the tools during work time [37,69]. Furthermore, organizations need to develop clear guidelines for their employees on how to use the eHealth tools at the workplace. Clear guidelines ensure a safe usage of these tools within the organization’s environment [70]. Guidelines can encompass recommendations regarding data security (eg, protection against unauthorized access or data transmission protection) or sharing information on social platforms (eg, recommendations on how to share health-relevant content safely) [53,70].

The organization benefits from having guidelines for the integration of eHealth tools. Table 1 presents organizational guidelines in each step of the implementation of eHealth tools in a WHP process. The guidelines are based on the proposed “life cycle model of Workplace Health Promotion” and can support the management and the steering group in responding to critical issues and can prevent an unsuccessful implementation of eHealth tools in WHP. The guidelines do not include the legal requirements for defining and implementing WHP in the countries or general guidelines to implement WHP that are not specifically related to eHealth tools. In Table 1, the most relevant references and an explanation or example are included for each action.

Security is of great importance for the successful integration of eHealth tools. Individuals have to be informed about the storage and usage of their individual data [12]. Privacy violations are possible from the technical point of view (eg, via hacking, outdated or nonexistent encryption methods, or legal interceptions) [12] or from the psychological view (eg, fears about data management and data reporting [71]). Even when reporting aggregated data (eg, arithmetical means of groups), attention should be paid that these data cannot be traced back to a specific person in the organization. This part can be tricky, as organizations want to have insight about the health status of their organization and thus of their employees. On the other hand, individual health data have to be protected strictly.

eHealth tools for WHP only can be successful if the employees trust the data management behind the tool. Particularly, the health domain is a very sensitive domain where violations of anonymity and privacy are experienced as harmful [81]. Invasions of privacy at the workplace undermine the trust in the tool and in the organization, which might lead to an unsuccessful WHP project.

**Table 1:** Guidelines for the organization for a safe usage of eHealth tools in workplace health promotion (WHP). Note: It is suggested to discuss all actions in the step “Concept/Adaptation” and find solutions before the WHP process starts.

Step, discussions and actions		Scientific base	Examples or explanation
**Concept/adaptation**			
	Privacy regulations of the employees’ health data	[71]	Privacy regulations of the employees’ health data have been discussed and suggestions have been included in the implementation concept. eHealth tools should be created in a way that prevent the steering group from seeing individual data
	Data security of the eHealth tool	[53,70]	Discussions with technical experts about protection against unauthorized access or data transmission protection
	Inclusion of all relevant persons in the WHP^a^ process	[38]	Next to the “traditional steering group,” technical experts or eHealth developers are included in the process as well
	Access to the WHP activities independent of Web and/or app access	[69]	Employees who do not have mobile phones should have access to the information (eg, via general accessible computers or by proving them with devices)
	Goals, added value, and benefits of the eHealth tool	[37]	Discuss in which steps of the process eHealth tools can give optimal support and where the traditional approach (without eHealth tools) is better suited
	Nondirective approach for using eHealth tools	[40]	Letting the employees themselves decide if and how to use the eHealth tools to avoid possible resistance
	Benefits, incentives	[72,73]	Incentives can help to enhance the signing up of the participants and help to keep the dropout rate at a low level
	Regulations about the usage of eHealth tools at the workplace	[37,69]	Provide opportunities to use the eHealth tools at work (eg, access to computers, mobile phones, and/or activity tracker), provide training and technical support
	Quality of the eHealth tools	[13,74]	eHealth tools that are used and integrated are chosen with regard to quality criteria in this area (eg, Mobile App Rating Scale, MARS [12] or enlight quality assessment and checklist [74])
**Information**				
	Information about privacy regulations and anonymity	[75]	Address all doubts, fears, and comments about privacy regulations and anonymity in the information process. Provide platforms where employees could voice their concerns and answer them adequately
	Usage for eHealth tools is on an opt-in base	[21]	The usage of any eHealth tools is free, employees can opt-in and are not obliged to use any tool
	Procedures to integrate the existing eHealth tools	[76]	Find solutions how to integrate the eHealth tools that are already used by the employees
	Definition of responsibilities in the process	[77]	A responsible person or a group is defined and introduced which serves as an expert(s) for the eHealth tool, and administers the process and is the “driver” for the process
**Assessment/analysis**			
	Execution and presentation of the assessment	[44]	It is suggested to use computers for more comprehensive assessments. If presenting on mobile devices, the questionnaires have to be adapted to fit the mobile phone screens
	Combination with other data sources	[76]	Combine questionnaire data with behavioral or psychophysiological data (eg, with the help of activity tracker) or with corporate key figures from the company	
	Data storage	[78]	Discuss the storage of data (eg, data have to be stored separately from e-mail addresses or other data that could be used to identify individuals)
**Dashboard feedback**			
	Content of the information provided on the dashboard	[31]	Discuss possibilities to personalize the dashboard content to the company’s needs
	Detail of the information provided on the dashboard	[31]	Specify a minimum number of entries for presenting results and subgroup analyses (eg, a minimum of 5 persons for a subgroup analysis) to avoid inference to a single person
	Regulations on how to share feedback information	[53,70]	Specify guidelines on how to share information on social platforms or other forums/platforms
	Inclusion of all relevant health experts	[57]	Health experts (eg, physicians, psychologists, sports experts, nutritionists) have been included in the interpretation of the results to avoid misinterpretations, and in the development of interventions
**Health circles/participatory planning**			
	Participation of employees in the selection of activities	[30]	All employees have been given the possibility to participate in the decision-making process to raise the employees’ acceptance toward the developed interventions and increase participation rates of employees
	Support of planning and organizing health circles	[79]	It includes “audience response systems” for discussions that allow employees to stay anonymous and see dashboard results immediately for a more fruitful discussion
**Interventions (individual/organization)**			
	Procedures for giving automated, individual feedback	[57]	The way of giving ethical, correct individual feedback to the employees is discussed and defined with health experts
	Procedures in case of critical results	[59]	A support line has been established in case employees need professional support after receiving a critical feedback
	Inclusion of organization-focused interventions	[21,26,61]	eHealth tools can support by providing management dashboards where the process and responsibilities are managed
**Evaluation**			
	Evaluation of the eHealth tool	[49,68]	A continuous improvement process is started where the evaluation results regarding the acceptance and usability of eHealth tool are addressed
	Evaluation of efficiency	[80]	Clear rationales and algorithms are found to monitor goals and effects with the support of eHealth tools

eHealth tools as well as processes regarding the implementation into the WHP process should be created together by developers, health experts, and WHP specialists to create an environment that is transparent, trustworthy, and safe for the employees. Including a multidisciplinary team (including all types of designers, stakeholders, supervisors, and end-users) already in an early phase of the development of the eHealth tool is a success factor for the further usage of these tools [38,68]. The tool should be designed in a way that it is easy to access and use [52]. In addition to including experts in the development phase, it is important to include relevant people in the implementation phase as well as in the evaluation phase of eHealth tools [82].

### The Important Role of Managers and Workplace Health Promotion Experts

WHP includes an interdisciplinary approach that combines different areas of expertise (eg, medicine, psychology, nutrition, safety) to achieve the most successful outcome [18,83]. Including all relevant people in the implementation process as well as including them during the whole WHP project can be a success factor for eHealth tools in WHP. Especially when using eHealth tools for individual-focused interventions (eg, tracking sports activities, monitoring nutrition intakes, or assessing health data), communicating with experts in the field of health promotion could motivate the participants to proceed with the intervention [44]. Using an unguided tool without human involvement could lead to nonparticipation, as important information and advices are not fully provided. Including social support in the form of peer mentors can be additionally included in the process to increase participation. Peer mentors can give advice or support healthy behavior with small incentives [84]. Including an incentive system to increase participation in eHealth programs could be a success factor for increasing participation as well [59,72].

At the workplace and especially for WHP, managers are important key factors that could influence the success of eHealth tools at work. A lack of support from the management is a major risk factor for the success of WHP projects [66]. Managers (in the top, middle, as well as in the lower management) are promoters of a healthy organizational climate and important key factors for successful and sustainable WHP activities [85,86]. In WHP projects, managers are able to positively influence employee health by supporting health promotion programs and policies and forwarding these policies to lower levels of management in a top-down process [87,88]. In addition, managers are responsible to provide resources for planning, implementing, and evaluating the WHP projects [89].

WHP programs have to be supported by the top management to demonstrate visibility and raise commitment of the developed WHP interventions [90]. In the WHP projects where managers do not actively promote the WHP programs, participants lose interest and are more likely to drop out [52]. Additionally, managers should provide all necessary resources to establish optimal conditions for the WHP projects [69]. To enhance the engagement of managers—and in the long run, the engagement of employees—the eHealth tools should be based on a scientific theory proving evidence of its benefits for the organization and its employees [37,91]. With a scientific theory behind, the mechanisms of change can be better assessed and evaluated [2].

The management is an important factor for the organization and implementation of WHP strategies, but is also a specific target group for WHP programs. eHealth tools can provide an added value for managers by supporting them in the field of leadership assistance and development [48].

## Conclusions

Integrating eHealth tools in WHP can be successful if they are integrated in all steps of the WHP process. They can enhance the efficiency of WHP projects with easier administration and management of the WHP process, providing an information and communication platform for all employees, and supporting the assessment of the current state of the organization. The clearest benefits lie in developing interventions for changing individual health behavior, as eHealth tools allow programming immediate, personal feedback that can be a strong reinforcement for behavior change. In addition to individual-focused interventions, organization-focused interventions can be supported by establishing electronic management dashboards that can be accessed everywhere at any time.

We presented guidelines that can help scientists and practitioners in successfully implementing eHealth tools in organizations. Some pilot studies were conducted in the practical field and provided first insights into where an eHealth tool can support the steering group and the employees during a WHP project.

The life cycle model of WHP can provide assistance for a successful implementation of eHealth tools in the WHP process. Ideally, a model like this can integrate the needs and obligations for psychosocial risk management [92], as the steps are very similar in the process (see also [19]). The assessment of psychosocial risks at the workplace is similar to WHP as both procedures aim to enhance health at the workplace. eHealth tools can support psychosocial risk management in the same way as the WHP processes.

Very clearly, the usage of these eHealth tools needs regulation and quality criteria as there are still many open questions such as data privacy and data security. At the moment, there is much uncertainty about the role of eHealth tools in organizations, especially when it comes to health-relevant data assessed within a WHP process. Good and clear directions for the usage and implementation of these tools are needed to secure quality and reach sustainable results. The developed guidelines for organizations can be the first step in supporting organizations to successfully implement eHealth tools in the WHP process.

## Abbreviations

eHealth: electronic health
mHealth: mobile health
WHO: World Health Organization
WHP: workplace health promotion
